# Amyloid deposition at the carotid artery in an ATTRwt amyloidosis patient: a case report

**DOI:** 10.1093/jscr/rjac567

**Published:** 2022-12-17

**Authors:** Hirokazu Ozaki, Nobuyuki Mitsui, Manabu Kinoshita, Mishie Tanino, Teruo Kimura

**Affiliations:** Department of Neurosurgery, Japanese Red Cross Kitami Hospital, Kitami 010-1406, Japan; Department of Neurosurgery, Asahikawa Medical University, Asahikawa 078-8510, Japan; Department of Neurosurgery, Japanese Red Cross Kitami Hospital, Kitami 010-1406, Japan; Department of Neurosurgery, Asahikawa Medical University, Asahikawa 078-8510, Japan; Department of Neurosurgery, Asahikawa Medical University, Asahikawa 078-8510, Japan; Department of Diagnostic Pathology, Asahikawa Medical University Hospital, Asahikawa 078-8510, Japan; Department of Neurosurgery, Japanese Red Cross Kitami Hospital, Kitami 010-1406, Japan

## Abstract

Systemic amyloidosis is a diseased condition where misfolded proteins deposit in various organs in the form of amyloids, and transthyretin deposition, termed ATTR amyloidosis, can be either an age-related amyloid formation from misfolded wild-type TTR (ATTRwt) or by hereditary TTR malfunction due to mutation in the TTR gene (ATTRv). Although ATTRwt amyloidosis can cause various diseases, such as cardiac failure, conduction disturbances, arrhythmias and carpal tunnel syndrome, it is still under-recognised considering its clinical significance. Here the authors report a case of ATTRwt amyloidosis leading to carotid stenosis requiring surgical intervention. To the best of our knowledge, the current report is the first that described histopathological evidence of amyloid deposition in the carotid artery due to ATTRwt amyloidosis.

## INTRODUCTION

Systemic amyloidosis refers to a diseased condition where misfolded proteins deposit in various organs in the form of amyloids [1]. Various precursors proteins are identified as the cause of amyloid formation leading to organ dysfunction, and among those precursors, transthyretin (TTR) protein is known to cause life-threatening organ dysfunction, termed ATTR amyloidosis [2]. ATTR amyloidosis can be either an age-related amyloid formation from misfolded wild-type TTR (ATTRwt) or by hereditary TTR malfunction due to mutation in the *TTR* gene (ATTRv) [3]. Although ATTRwt amyloidosis can cause various diseases, such as cardiac failure, conduction disturbances, arrhythmias and carpal tunnel syndrome, it is still under-recognised, considering its clinical significance with increasing prevalence in the aged population [4]. Here the authors report a case of ATTRwt amyloidosis leading to carotid stenosis requiring surgical intervention. To the best of our knowledge, the current report is the first that described histopathological evidence of amyloid deposition in the carotid artery due to ATTRwt amyloidosis.

## CLINICAL SUMMARY

**Figure 1:**
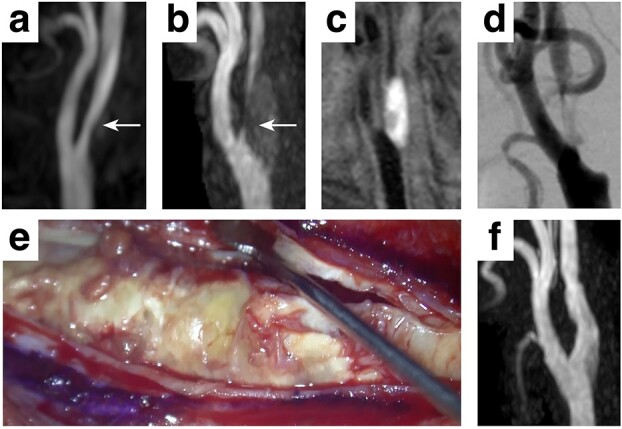
Clinical presentation A mild stenosis at the right carotid artery (white arrows, **a**) progressed in 3 years (white arrows, **b**), requiring surgical intervention. The carotid plaque showed a high signal intensity on T1WI (**c**) with 93% stenosis assessed by the NASCET criteria (**d**). A standard carotid endarterectomy was performed (**e**) without any complication (**f**).

A 77-year-old Japanese male with a past medical history of acute cardiac infarction was diagnosed with a right carotid artery stenosis which progressed in three years (Fig. 1a and b, white arrows). The carotid plaque showed a high signal intensity on the T1-weighted image (T1WI), suggesting a vulnerable plaque containing intraplaque hemorrhage (Fig. 1c). A digital subtraction angiogram revealed a 93% internal carotid artery stenosis according to the North American Symptomatic Carotid Endarterectomy Trial (NASCET) starting at the carotid bifurcation (Fig. 1d). The patient was elected for carotid endarterectomy rather than carotid artery stenting, considering the surgical risks accompanying the presumed vulnerable plaque [5]. However, during the presurgical assessment, an echocardiogram showed a decreased left ventricular ejection fraction accompanied by bright myocardium and left atrial dilatation, suspicious of cardiac amyloidosis. Myocardial pathological examination by a myocardial biopsy achieved a final diagnosis of ATTRwt amyloidosis. Carotid endarterectomy was performed uneventfully, and the patient was discharged 9 days after surgery with a modified Rankin Scale 0 (Fig. 1f).

## PATHOLOGICAL FINDINGS

**Figure 2:**
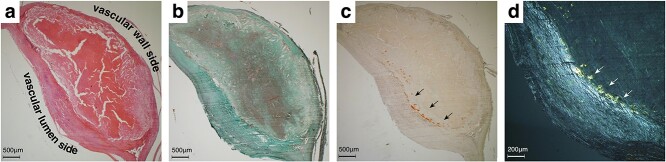
Histological and immunohistochemical findings. Hematoxylin and eosin staining revealed carotid intima’s eccentric fibrosis (**a**). Masson’s trichrome staining showed a neovascularised plaque surrounded by smooth muscle fibers (**b**). Potassium permanganate (KMnO4)-DFS stain showed positive (black arrows, **c**), and the deposits were apple-green-birefringence positive (white arrows, **d**).

The hematoxylin and eosin-stained surgical specimen revealed carotid intima’s eccentric fibrosis, compatible with arteriosclerotic change (Fig. 2a). Masson’s trichrome staining showed that the plaque consisted of fibers surrounding scattered smooth muscle cells and neovascularisation (Fig. 2b). Positive staining was observed on potassium permanganate (KMnO4)-DFS stain (Fig. 2c), and the stained deposits were apple-green-birefringence positive (Fig. 2d), consistent with a non-AA amyloid deposition at the carotid artery [6, 7].

## DISCUSSION

Amyloid cardiomyopathy refers to the myocardial deposition of amyloid fibers causing cardiac dysfunction, and it comprises AL (also known as primary) amyloidosis and ATTR amyloidosis [1]. Although these two types of amyloid cardiomyopathy share similar clinical presentations, the underlying mechanisms differ. AL amyloidosis is caused by tissue deposition of immunoglobulin light-chain amyloid, whereas ATTR amyloidosis is caused by either wild-type or variant transthyretin tissue deposition [2, 8]. AL amyloidosis was historically considered the most frequent type of amyloidosis that is untreatable at symptom onset. However, chemotherapy aiming to suppress immunoglobulin-producing plasma cells has recently proven effective [9].

On the other hand, ATTR amyloidosis was considered a much infrequent rare condition compared with AL amyloidosis. However, extensive research has been pursued on ATTR amyloidosis due to its clinical significance in heart failure and it has recently been reported that amyloid deposits derived from plasma transthyretin are present in the heart of up to 25% of elderly individuals [10]. Tafamidis, a benzoxazole derivative that binds to the thyroxine-binding sites of transthyretin, was recently shown to exhibit clinical benefit to transthyretin amyloid cardiomyopathy, changing the treatment paradigm of ATTR amyloidosis [11]. In addition to its higher-than-expected prevalence, the newly developed ATTR amyloidosis targeting agent makes it clinically more relevant than ever to correctly identify and diagnose ATTR amyloidosis patients. Thus, there is concern that many ATTRwt amyloidosis patients are under-recognised and not receiving optimal treatment. Although amyloidosis can possibly cause carotid stenosis [12], the presented case pathologically showed, for the first time, that carotid stenosis can represent amyloidosis and that great suspicion should be taken for senile patients [13]. As ATTRwt amyloidosis can be correctly diagnosed under great suspicion of these, we suggest that pathological examination looking for amyloid deposits should be performed for patients who have undergone carotid endarterectomy. Likewise, cardiac examinations considering amyloidosis should be performed for those elected for carotid stenting.
